# Four-Dimensional Characterization of Thrombosis in a Live-Cell, Shear-Flow Assay: Development and Application to Xenotransplantation

**DOI:** 10.1371/journal.pone.0123015

**Published:** 2015-04-01

**Authors:** Donald G. Harris, Prabhjot K. Benipal, Xiangfei Cheng, Lars Burdorf, Agnes M. Azimzadeh, Richard N. Pierson

**Affiliations:** 1 Division of General Surgery, Department of Surgery, University of Maryland School of Medicine, Baltimore, Maryland, United States of America; 2 Center for Vascular and Inflammatory Diseases, University of Maryland School of Medicine, Baltimore, Maryland, United States of America; 3 Division of Cardiac Surgery, Department of Surgery, University of Maryland School of Medicine, Baltimore, Maryland, United States of America; 4 Surgical Care Clinical Center, VA Maryland Health Care System, Baltimore, Maryland, United States of America; BloodCenter of Wisconsin, UNITED STATES

## Abstract

**Background:**

Porcine xenografts are a promising source of scarce transplantable organs, but stimulate intense thrombosis of human blood despite targeted genetic and pharmacologic interventions. Current experimental models do not enable study of the blood/endothelial interface to investigate adhesive interactions and thrombosis at the cellular level under physiologic conditions. The purpose of this study was to develop and validate a live-cell, shear-flow based thrombosis assay relevant to general thrombosis research, and demonstrate its potential in xenotransplantation applications.

**Methodology/Principal Findings:**

Confluent wild-type (WT, n = 48) and Gal transferase knock-out (GalTKO, which resist hyperacute rejection; n = 11) porcine endothelia were cultured in microfluidic channels. To mimic microcirculatory flow, channels were perfused at 5 dynes/cm^2^ and 37°C with human blood stained to fluorescently label platelets. Serial fluorescent imaging visualized percent surface area coverage (SA, for adhesion of labeled cells) and total fluorescence (a metric of clot volume). Aggregation was calculated by the fluorescence/SA ratio (FR). WT endothelia stimulated diffuse platelet adhesion (SA 65 ± 2%) and aggregation (FR 120 ± 1 a.u.), indicating high-grade thrombosis consistent with the rapid platelet activation and consumption seen in whole-organ lung xenotransplantation models. Experiments with antibody blockade of platelet aggregation, and perfusion of syngeneic and allo-incompatible endothelium was used to verify the biologic specificity and validity of the assay. Finally, with GalTKO endothelia thrombus volume decreased by 60%, due primarily to a 58% reduction in adhesion (P < 0.0001 each); importantly, aggregation was only marginally affected (11% reduction, P < 0.0001).

**Conclusions/Significance:**

This novel, high-throughput assay enabled dynamic modeling of whole-blood thrombosis on intact endothelium under physiologic conditions, and allowed mechanistic characterization of endothelial and platelet interactions. Applied to xenogeneic thrombosis, it enables future studies regarding the effect of modifying the porcine genotype on sheer-stress-dependent events that characterize xenograft injury. This in-vitro platform is likely to prove broadly useful to study thrombosis and endothelial interactions under dynamic physiologic conditions.

## Introduction

Pig to human xenotransplantation is a potential means of addressing the critical shortage of organs available for transplantation.[1–4] However, human antibodies against the porcine galactose 1,3α-galactose (Gal) antigen and subsequent complement activation trigger endothelial injury and thrombosis, resulting in hyperacute rejection of wild type (WT) porcine organs.[1,4–10] Organs from pigs that do not express Gal (GalTKO) have significantly improved survival,[1,4,11–13] which is further enhanced by transgenic expression of human proteins, such as the complement regulatory protein CD46 (GalTKO.hCD46). [14] Despite these advances, thrombosis remains a critical process associated with xenograft injury.[2,4,12,13,15–20] Current strategies to control acute thrombosis include adding human thromboregulatory transgenes, such as for endothelial protein C receptor, to the existing genetic background (GalTKO.hCD46.hEPCR).[1,4,21] As such, the ability to mechanistically characterize thrombus formation is critical to studying the effects of genetic and pharmacologic interventions on xenograft injury.

Previously described models used for xenotransplantation and general thrombosis research have limited ability to investigate the mechanisms contributing to thrombotic xenograft injury under physiologic conditions. While *ex*- and *in-vivo* whole organ studies are valuable and clinically translatable models of pig-to-human transplantation, they involve multiple incompletely understood pathways, and lack the cellular resolution to dynamically study events occurring at the blood—endothelial interface.[11–13,15,22] Conversely, static *in-vitro* assays lack physiologic shear-flow,[10,23–26] which is a critical condition for a range of interactions occurring at the endothelial interface,[27–30] including many involved in thrombus formation, stabilization and resolution.[27,29,31–35]

In contrast, models utilizing shear-flow enable dynamic study of thrombosis under reproducible and controlled physiologic conditions.[23,36] However, standard *in-vitro* perfusion models typically use ligand-coated surfaces rather than confluent endothelium,[23,31,33,37–40] limiting translation to *in-vivo* conditions and providing no ability to study endothelial interactions. Those studies that do utilize live-cell endothelia under shear flow have typically relied on intra-vital microscopy,[36,41–43] which increases resource use, hinders throughput and limits the ability to control experimental conditions.

A cellular *in-vitro* shear-flow platform has not previously been used to study thrombosis. Such a system potentially combines the biologic relevance of physiologic flow over living endothelium, similar to perfused tissue in whole organ and animal models, but with the additional advantages of scalability, reproducibility, experimental control and minimal resource requirements that are characteristic of shear-flow models. The purpose of this study was to develop and validate a novel perfusion assay that models thrombus formation on endothelium over time and under physiologic shear flow conditions. Here we demonstrate its initial application in studying the highly thrombotic conditions encountered in our xenotransplantation research.

## Methods

### Ethics statement

The University of Maryland, Baltimore Animal Care and Use Committee approved all animal procedures, which were performed in accordance with National Institute of Health guidelines. All human blood samples were obtained from a single consenting volunteer member of the research team (DGH). Institutional Review Board approval was not in place at the time of research, but approval to publish the data was retrospectively granted after an IRB audit and procedural review.

### Instrumentation

Experiments were performed using a Bioflux 1000 system (Fluxion Biosciences, South San Francisco) coupled with an automated AxioObserver Z1 microscope (Zeiss, Oberkochen).[44,45] 24 channel low-shear microfluidic plates (Fluxion) were used. Each channel consists of a proximal inflow well that flows via a short segment to the perfusion chamber (length 4.8 mm, width 0.35 mm, height 0.07 mm), which subsequently flows to an outflow well. Shear-flow is generated by pneumatic pressure delivered to the inflow well; perfusate does not recirculate.[44,45] A plate holder with an electric heating element was used for all experiments. Perfusion and imaging sequences were controlled using system software from a standard desktop computer.

### Cell culture

Wild type and GalTKO tissue culture adapted porcine aortic endothelial cell (PAEC) lines (14259 and 15502, respectively) were used for assay development. To validate the findings, the experiments were repeated using primary WT and GalTKO.hCD46 PAEC; the latter were used because no primary GalTKO PAEC were available to us when these experiments were performed, and WT and GalTKO.hCD46 genotypes have comparable platelet consumption and thrombin generation profiles in whole-lung xenoperfusion experiments. [14] Finally, to test the assay system under non-thrombogenic, non-xenogenic reference conditions (to provide a “negative control”), and for “allo-incompatible” porcine—porcine studies, GalTKO.hCD46.hEPCR (the animal phenotype available at that time) PAEC and blood were used.

Porcine thoracic aortae were removed after heart-lung explantation and used for primary porcine aortic endothelial cell (PAEC) cultures as previously described.[11,46] Briefly, WT, GalTKO.hCD46 and GalTKO.hCD46.hEPCR PAECs were isolated by mechanical scrapping and seeded in 0.5% gelatin (Sigma-Aldrich, St. Louis) coated culture flasks. Standard culture medium for all conditions consisted of Dulbecco’s modified Eagles medium containing 1 g/L D-glucose, 110 mg/L sodium pyruvate and L-glutamine (Gibco—Life Technologies, Grand Island), and supplemented with 10% heat-inactivated fetal bovine serum (Atlanta Biological, Lawrenceville), gentamicin, amphotericin B. Endothelial cell growth supplement (Becton Dickinson, San Jose) was used at 100 μg/mL for first-passage primary PAECs and 50 μg/mL for all other cultures. Cells were grown in 75 cm^2^ flasks, and split approximately every 3 days. Endothelial morphology was verified at each passage. Cells were used at the 4^th^ to 8^th^ passage by harvesting via trypsinization (0.25%; Gibco) and suspension at 10 million/mL in medium. Standard incubation conditions for culture flasks and plated monolayers were 37°C and 5% CO_2_.

### Flow channels

Channels were coated with 100 μg/mL bovine plasma fibronectin (Sigma-Aldrich) in phosphate buffered solution (PBS) by perfusion at 5 dynes/cm^2^ (shear rate 125/s) for 5 minutes. After setting for 1 hour at room temperature, the channels were washed with culture medium at 5 dynes/cm^2^ for 10 minutes. Harvested PAECs were seeded by loading 40 μL of cell suspension into the inflow wells. A PAEC bolus was perfused at 0.5 dynes/cm^2^ into the channels, followed by a 60 second pause to allow initial attachment. Additional boluses were delivered to concentrate the cells to approximately 100–150 per 100x field of view (S1 Fig.).

The plate was incubated for 1 hour to achieve firm PAEC attachment and spreading. 800 μL of medium was introduced to the outflow wells to nourish the channels by gravity flow. The excess PAECs in the inflow wells were removed by two cycles of washing with 400 μL sterile deionized water and incubation with 150 μL 0.25% trypsin for 5 minutes. The plate was incubated for 48–72 hours, and the channels were used at 100% confluence as confirmed by brightfield microscopy. Excess medium was aspirated from inflow and outflow wells immediately prior to experiments.

### Perfusates and perfusions

Xenoperfusion experiments on porcine endothelia were performed with fresh human whole blood. Small volume (4–8 mL) samples were collected by venipuncture using sodium heparin coated vacutainers (75 units/4mL; Becton Dickinson, Franklin Lakes). To evaluate the role of anti-Gal antibodies in thrombosis under non-xenogenic “allo-incompatible” conditions, blood from GalTKO.hCD46.hEPCR pigs (which contains ‘natural’ anti-Gal antibodies) was perfused over Gal-expressing WT PAEC endothelium. Blood and PAECs from clonally-derived GalTKO.hCD46.hEPCR pigs were used to provide a syngeneic blood-EC condition, to serve as a non-thrombogenic biologic reference condition (“negative control”) during assay development.

Blood aliquots were fluorescently labeled with 0.5 μM calcein-AM (Becton Dickinson) to stain leukocytes and platelets, and treated with 0.5 μg/mL abciximab (Eli Lilly, Indianapolis), 0.2 μg/mL bivalirudin (Hirulog; The Medicine Company, Cambridge) or PBS vehicle. Aliquots were light-protected and mixed by rocking for 30 minutes at room temperature. High-dose anticoagulation was verified by pre-perfusion activated clotting times (ACT Plus system with HR-ACT cartridges; Medtronic, Minneapolis), and physiologic blood cell counts were confirmed by an automated counter (Hemavet; Drew Scientific, Waterbury).

350 μL of prepared blood perfusate was introduced into the inflow wells of experimental channels. To model physiologic, low-resistance flow such as encountered in the microcirculation or pulmonary vasculature, blood was perfused at 5 dynes/cm^2^ and 37°C for 50 minutes.[29,47,48] The most proximal 100x field of view of each channel’s perfusion chamber was imaged every 30 seconds by fluorescent microscopy; a 100 ms exposure was used to obtain a robust signal from adhered thrombi and enable identification and filtration of streak artifacts. Typically, 12 channels were perfused and acquired simultaneously: 2 positive controls, 5 study controls and 5 experimental channels.

### Data analysis

The sequential source images were stacked for their respective channels and analyzed using a component of Montage (Fluxion Biosciences) based on the Metamorph engine (Molecular Devices, Sunnyvale). To capture the most biologically and physiologically significant event within each channel, the signal threshold for each stack was determined from the image with the highest gross thrombosis. Thresholds were highly consistent within and between experiments. Fluorescent intensity (in arbitrary units, a.u.) and surface area coverage (SA, percent) and were extracted from image stacks and exported for data analysis.

As fluorescence is a function of the labeled platelet mass, it was used as a measure of total thrombus volume (TV, in arbitrary units).[33] SA was applied directly as a measure of adhesion in the *x* and *y* dimensions between the endothelium and perfused platelets.[40] The fluorescence:SA ratio (FR, a.u.) determined relative binding in the *z* dimension, and was used as an index of platelet aggregation.[33] Binding kinetics were measured by the time to 50% peak SA (T_50_, minutes). Mean peak TV, SA, FR and T_50_ values were compared by unpaired Student’s *t*-test. To correct for the well-described artifact of photobleaching, when analyzing serial time points a constant total fluorescence value was manually maintained for subsequent frames if fluorescence became degraded in the setting of visibly unchanged thrombus volume. Results were qualitatively assessed by three-dimensional surface renditions using ImageJ (National Institutes of Health, Bethesda).[49]

## Results

**Fig 1:**
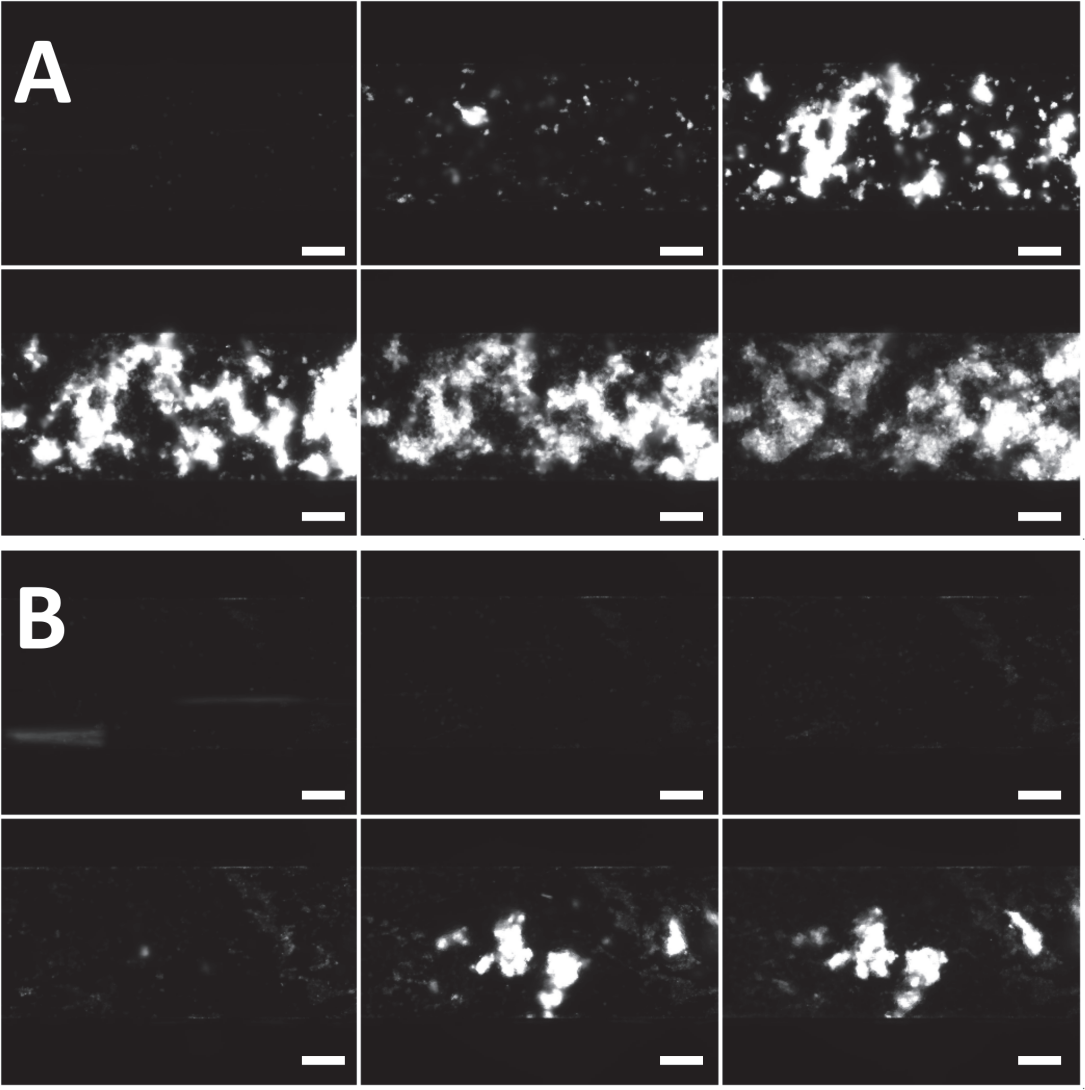
A & B. Representative xenothrombosis on wild type and GalTKO endothelia. Sequential source images of a single A) WT and B) GalTKO cell line endothelia perfused with heparinized human blood taken at 0, 10, 20, 30, 40 and 50-minute intervals (left-to-right by row). Panel A demonstrates the typical high-grade thrombosis stimulated by WT endothelium, with early and diffuse adhesion and aggregation. In Panel B, the GalTKO genotype reduces and delays adhesion, resulting in less overall thrombosis. However, relative aggregation remains intact where thrombus is present. Images are at 100x magnification using a 100-millisecond exposure. Scale bars: 100μm.

**Fig 2:**
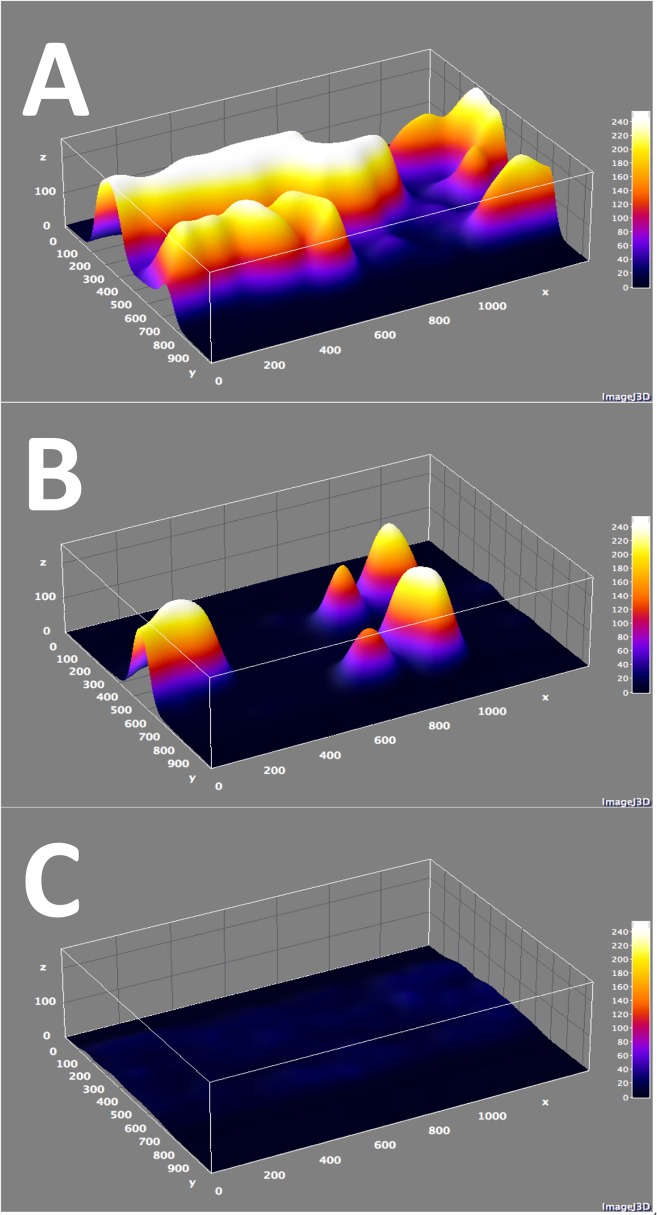
A—C. Representative volumetric thrombus models. 3D surface renderings of end-perfusion images (*t* = 50 minutes) for WT and GalTKO endothelia perfused with heparinized human blood (A & B, respectively) and WT endothelium perfused with heparinized human blood treated with 0.5 μg/mL abciximab (C). Panel A demonstrates significant adhesion and aggregation. In Panel B adhesion is reduced but aggregation is mostly intact, while in Panel C aggregation is ablated. Source images were taken at 100x magnification using a 100-millisecond exposure and rendered using ImageJ.

Blood samples selected for analysis had physiologic platelet and cell counts and consistent anticoagulation with activated clotting times > 999 sec (S1 and S2 Tables). Initial experiments were performed by perfusing WT 14259 cell line endothelia with heparinized human blood to recapitulate hyperacute xenograft injury mediated by anti-Gal antibodies, and develop a reference condition for maximal thrombosis to serve as a positive control (Fig. 1A & S1 Video). Despite high-dose heparin anticoagulation, WT endothelia rapidly stimulated diffuse adhesion and aggregation, indicating consistent, high-grade thrombosis (Table 1). 3D surface renditions correlated with these findings (Fig. 2A), and when sequenced, enabled dynamic four-dimensional representation of thrombosis (S2 Video). Consistent with these initial results, primary WT cell line endothelia stimulated significant thrombosis (S3 Table). However, primary WT PAECs resulted in even more rapid thrombosis and obstruction of the proximal channel; loss of flow due to rapid channel occlusion presumably accounts for significantly lower overall adhesion and total thrombus volume in assays using WT primary PAECs (S3 Table).

**Table 1:** Results from xenoperfusion of porcine endothelia with human blood.

Cells:	WT	GalTKO	WT	WT
**Perfusate:**	human blood	human blood	human blood	human blood
Treatment:*	none	none	abciximab	bivalirudin
**N**	48	11	5	9
**Thrombus Volume (TV), a.u.**	7831 ± 238	3098 ± 594	822 ± 137	2999 ± 891
Δ		-60%	-89%	-62%
*P*		<0.0001	<0.0001	<0.0001
**Adhesion (SA), %**	65.0 ± 1.7	27.5 ± 4.2	49.6 ± 4.1	25.4 ± 7.0
Δ		-58%	-24%	-61%
*P*		<0.0001	0.04	<0.0001
**Aggregation (FR), a.u.**	120.0 ± 1.0	107.0 ± 4.9	16.2 ± 1.8	112.7 ± 3.3
Δ		-11%	-87%	-6%
*P*		<0.0001	<0.0001	0.01
**Kinetics (T**_**50**_ **), min.**	21.9 ± 1.7	31.0 ± 2.8	22.4 ± 2.2	29.8 ± 5.4
Δ		+42%	+2%	+36%
*P*		0.02	0.93	0.10

*All experiments were performed with heparinized blood. a.u., arbitrary units; TV, thrombus volume; Δ, relative change; SA, percent surface area coverage; FR, fluorescence ratio; T_50_, time to 50% maximal surface area coverage. All Δ & *P* values are versus WT controls, and are expressed as mean ± SEM.

To exclude platelet and coagulation activation by the system itself, post-perfusion images of the inflow reservoirs upstream from the PAEC endothelia were obtained. No significant thrombi were present in device inflow chambers; instead thrombus formation was limited to the endothelium in the perfusion channel itself (S2 Fig.). Similarly, perfusion of GalTKO.hCD46.hEPCR porcine blood on syngeneic endothelia (from cloned pigs) yielded negligible thrombosis (Table 2), indicating that the BioFlux system is not intrinsically thrombogenic, and that the thrombosis during xenoperfusion was the result of specific interactions between the human blood and porcine endothelium. In contrast, Gal- expressing WT endothelia perfused under “allo-incompatible” conditions with GalTKO.hCD46.hEPCR porcine blood (in which anti-Gal antibodies are present, as with during xenoperfusion) stimulated intense thrombosis, similar to WT PAEC-human blood positive controls. Together, these results indicate the system conditions are not inherently thrombogenic, and that the thrombus formation is instead a specific function of the biologic interactions between the perfusate and the endothelium.

**Table 2:** Results from perfusing porcine endothelia with syngeneic and allo-incompatible porcine blood.

Cells:	WT	GalTKO.hCD46.hEPCR
**Perfusate:**	GalTKO.hCD46.hEPCR blood	GalTKO.hCD46.hEPCR blood
Treatment:*	none	none
**N**	4	6
**Thrombus Volume (TV), a.u.**	8259 ± 1074	466 ± 68
Δ		-94%
*P*		<0.0001
**Adhesion (SA), %**	67.2 ± 8.4	17.3 ± 2.7
Δ		-74%
*P*		0.0003
**Aggregation (FR), a.u.**	122.4 ± 1.1	28.4 ± 3.7
Δ		-77%
*P*		<0.0001
**Kinetics (T**_**50**_ **), min.**	29.5 ± 6.9	26.6 ± 5.5
Δ		-10%
*P*		0.76

*All experiments were performed with heparinized blood. a.u., arbitrary units; TV, thrombus volume; Δ, relative change; SA, percent surface area coverage; FR, fluorescence ratio; T_50_, time to 50% maximal surface area coverage. All Δ & *P* values are versus WT controls, and are expressed as mean ± SEM.

**Fig 3:**
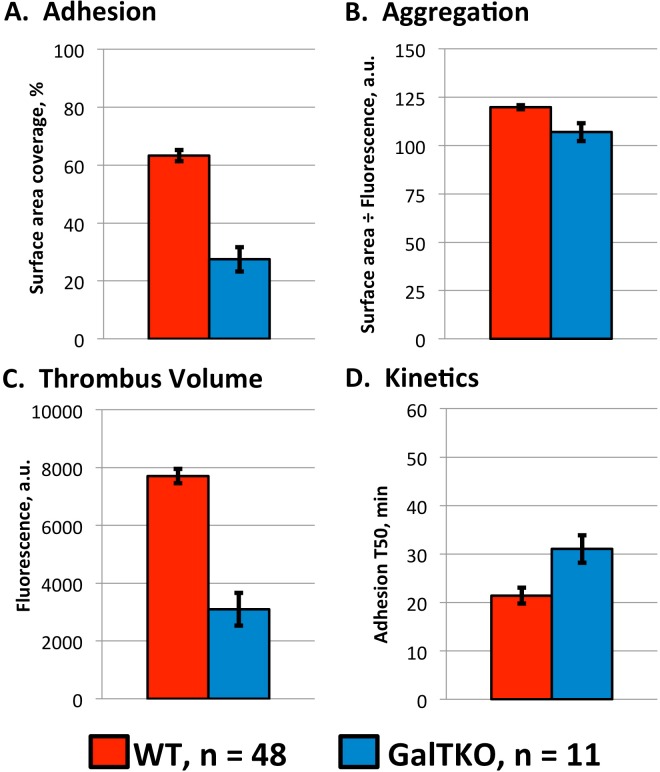
A—D. Mechanistic parameters of wild type versus GalTKO thrombosis. Summary of the effects of the GalTKO genotype on xenogeneic thrombosis in comparison to WT endothelia. Adhesion is substantially reduced (Panel A), so despite relatively intact aggregation (B), overall thrombus volume is reduced (C). Thrombosis was also delayed by the GalTKO genotype (D). *: *P* < 0.0001, **: *P* < 0.01. Values are expressed as mean ± SEM.

**Fig 4:**
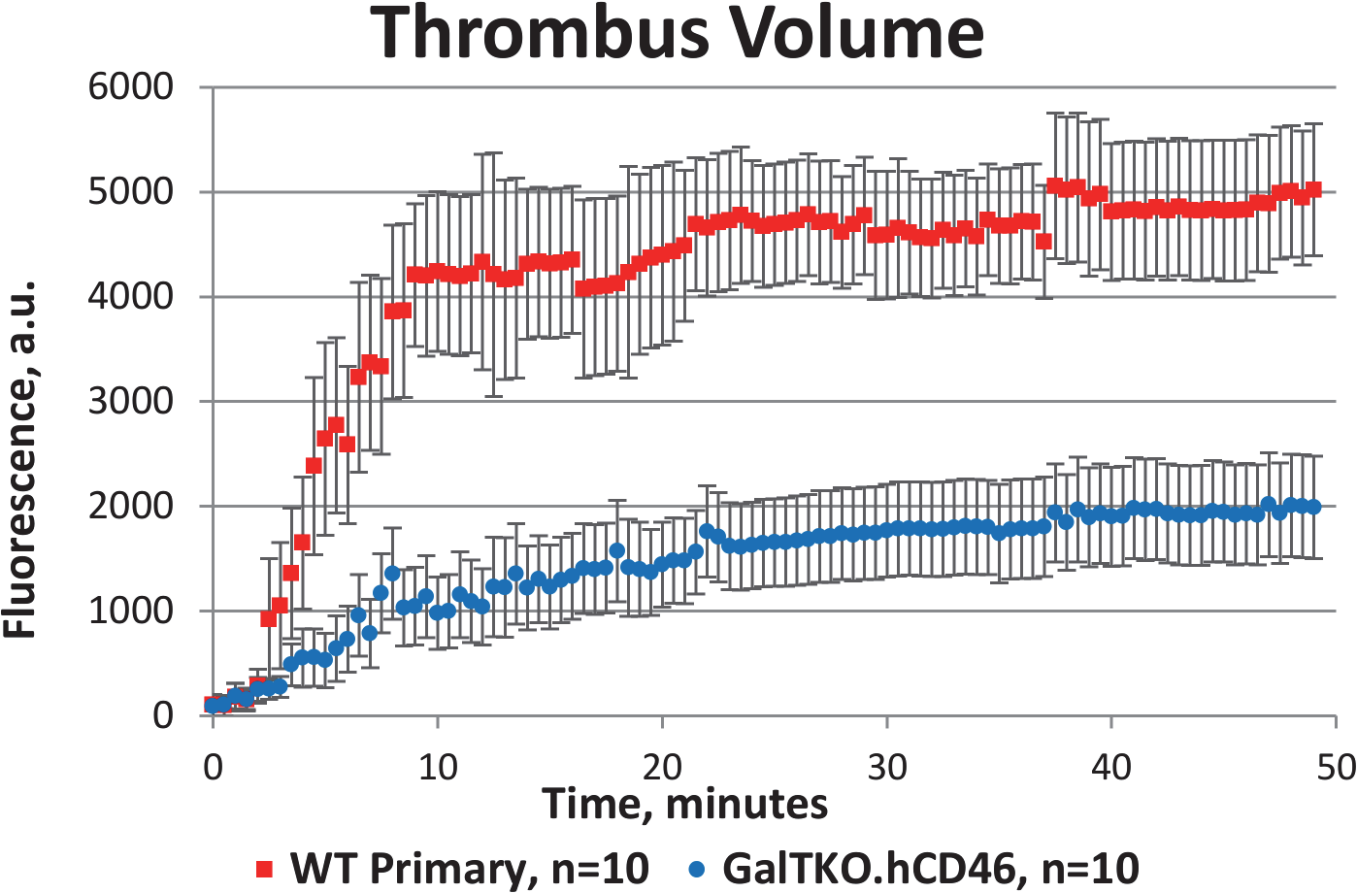
Thrombus generation curves for primary wild type and GalTKO endothelia. Summary of thrombus volume for primary WT versus GalTKO.hCD46 endothelia perfused with heparinized human blood. a.u., arbitrary units. Values are corrected for streak artifact (presumed embolization events) and photo-bleaching, and are expressed as mean ± SEM.

To validate the assay by correlation with established xenotransplantation findings, GalTKO endothelia were tested in comparison to WT controls (Fig. 1B, S3 and S4 Videos). Consistent with the improved physiologic performance and extended survival of GalTKO relative to WT xenografts in existing whole-organ xenoperfusion models, thrombosis was suppressed and delayed on GalTKO 15502 cell line endothelia (Table 1). This effect was primarily attributable to diminished surface area adhesion, as the intensity of aggregation was only minimally affected in areas where thrombus formation was present (Figs. 2B and 3). Primary GalTKO.hCD46 endothelial cultures yielded similar results, demonstrating that this result is not an artifact of the GalTKO cell line used (Fig. 4 & S3 Table).

Because thrombus propagation is dependent on glycoprotein (GP) IIbIIIa-mediated platelet—platelet binding and consequent aggregation,[50] the utility and validity of FR as a measure of thrombus propagation was explored. WT endothelia were perfused with whole human blood treated with abciximab, an anti-GPIIbIIIa Fab.[51] Consistent with specific inhibition of platelet—platelet interactions, abciximab had only a minor effect on SA and T_50_, but substantially reduced FR and subsequently TV (Table 1, Fig. 2C). In contrast, bivalirudin, a direct thrombin inhibitor that interrupts coagulation cascade amplification but not platelet aggregation, decreased SA but not FR, indicating no specific effect on aggregation (Table 1).

## Discussion

We describe validation of a novel cellular perfusion assay that enables dynamic and mechanistic characterization of thrombus formation on the endothelial surface under physiologic *in-vitro* conditions. Consistent with the results and kinetics from established xenotransplantation models,[10,11] WT porcine endothelia rapidly stimulated intense thrombosis of human blood despite high-dose anticoagulation. Thrombus formation occurred with kinetics similar to the thrombin formation and platelet activation and sequestration reported by our group during *ex-vivo* WT lung xenoperfusion, suggesting that the BioFlux model accurately simulates ex vivo whole organ perfusion.[11] Overall thrombosis was decreased and delayed on GalTKO and GalTKO.hCD46 endothelia relative to WT cell lines and primary endothelia; again this observation correlates with the improved survival seen using pig lungs with these genotypes in published in vivo lung transplant experiments and in our *ex-vivo* lung xenograft perfusion model.[11–13] Experiments with syngeneic pig blood confirmed that these findings were the specific result of interactions between porcine endothelia and the perfused human blood rather than an artifact of the system itself, and confirmed that thrombosis is initiated by anti-Gal antibody under non-xenogenic ‘allo-incompatible’ conditions.

This assay incorporates conditions that simulate physiologic aspects of *in-vivo* thromboregulation, including live-cell endothelium, whole blood perfusate, and adjustable physiologic shear-flow conditions. Together, these features provide a potentially useful platform to model the endothelial—blood interactions occurring in the low-resistance capillaries that account for the bulk of such interactions in native, transplanted, or *ex-vivo* perfused organs. Therefore, we conclude that this assay is suitable for both definitive *in-vitro* studies, and as a platform for hypothesis-testing before proceeding to more resource intensive (and mechanistically cumbersome) *ex-vivo*, *in-vivo*, or *intra-vital* experimental models.

While the primary purpose of this project was to develop and validate the assay for thrombosis research, interestingly, the model demonstrated its potential utility by showing for the first time that reduced thrombosis on GalTKO endothelia was primarily due to decreased and delayed platelet adhesion; platelet aggregation was only marginally affected by the GalTKO phenotype, and platelet accumulation remained largely intact in areas where visually appreciable initial adhesion occurred. Although the GalTKO genotype is associated with decreased thrombosis in other organ xenograft models, [11,13,17] this study is the first to define the process mechanistically. This new finding supports strategies to control platelet adhesion in xeno models, which might include preventing ‘non-physiologic’ binding between human GPIb and porcine von Willebrand factor. Our observations also support our current working hypothesis that coagulation pathway activation and a variety of cell adhesive interactions will likely need to be targeted by future genetic and pharmacologic interventions to minimize platelet aggregation and clot formation in pig-to-human organ xenografts.[15,20,52–55]

This assay has multiple immediate applications in the xenotransplantation field. It enables efficient screening of candidate pig genotypes and comparative testing of anticoagulation regimens. This platform can be adapted to evaluate endothelial pre-treatment or conditioning prior to xenoperfusion, which may enable more rapid development and testing of candidate transplantation regimens prior to application in animal models.

Importantly for xenotransplantation research, accelerated thrombosis with primary WT endothelia relative to PAEC cell lines presumably reflects loss of prothrombotic properties associated with immortalization or multiple passaging. By inference, data acquired using cell line PAEC may *under*estimate thrombotic potential. More generally, by verifying and demonstrating characterization of thrombosis on cell lines as well as primarily derived endothelial cells, and under non-xenogenic ‘allo-incompatible’ conditions, these experiments serve to validate this model as a platform for the mechanistic study of thrombus formation and resolution.

The assay has important biologic and technical limitations. First, the fibronectin/PAEC flow chamber construct recapitulates only the xenograft endothelium: while suitable for modeling processes occurring at the endothelial surface, the lack of physiologic native subendothelial matrix and intimal cell layers, which normally contribute to physiologic flow regulation and may modulate luminal thrombosis after endothelial loss or injury, may limit the utility of the assay for some applications. Further, although individual endothelial—platelet interactions and microenvironment contribute to cellular activation and platelet adhesion,[36,56,57] these were not specifically assessed in this assay. Unlike other models that measure actual or estimated absolute thrombus volume, this assay provides relative volume and aggregation measures based on fluorescent intensity. Assay sensitivity and quantitative accuracy may be limited by dye quenching for experiments using long exposure times and/or frequent imaging. However, achieving reproducible volumetric measurements is nevertheless feasible by using consistent methods and techniques.

Although useful for characterizing and validating this method of visualizing and quantified thrombosis, the experimental differences necessitated by the limitations of available of PAEC and pig blood for primary cell and non-xenogenic perfusions limits comparison between the genotypes. It is conceivable that expression of hCD46 or hEPCR may have influenced our observations in unexpected ways, but the adding hCD46 to the GalTKO background has minimal effect on platelet consumption or thrombin generation in xenoperfused pig lungs. [14] Indeed, our data supports the prediction that hCD46 expression would not significantly influence direct PAEC interaction with human platelets or coagulation pathway components. Finally, while hEPCR (in the context of GalTKO.hCD46.hEPCR PAECs) is expected to have direct thromboregulatory activity in pig-to-human *xeno*transplantation, no confounding effect of hEPCR expression is expected in *syngeneic* or allo-incompatible experiments. Regardless of the specific genotype, under syngeneic conditions, compatible blood and endothelium is not thrombotic, while during allo-incompatible perfusion of blood from GalTKO pigs or their derivatives intense thrombosis is attributable to the circulating anti-Gal antibodies as with xenoperfusion of human blood on WT endothelium.

## Conclusion

This novel, cellular assay enabled high-throughput and dynamic modeling of whole-blood thrombosis on intact endothelium under physiologic conditions, with definition of the mechanistic components of thrombus formation. For xenotransplantation, it is a potentially valuable new method to study processes contributing to xenograft injury at the interface between porcine EC’s and human blood. By enabling dynamic and mechanistic characterization of thrombus formation under shear flow, it offers the potential to provide insight into the effects of genetic and pharmacologic manipulation on xenogeneic thrombosis. As such, we predict it will become a valuable platform for future xenotransplantation studies, and prove generally useful in thrombosis research.

## Supporting Information

S1 Fig Summary of endothelial preparation. Endothelial cells are perfused from the inflow well (A) into the channel, where they are cultured to confluence over 48–72 hours (B & D). Scale bars: 100μm.
(TIF)

S2 Fig End-perfusion images. After 50 minutes of perfusion, blood remaining in the inflow well reservoir demonstrates no significant thrombosis (A), which is instead limited to the perfusion channel (B).
(TIF)

S1 Table Summary of complete blood counts. Results were obtained using a Drew Scientific Hemavet automatic counter. WBC: white blood cells; Hgb: Hemoglobin; Hct: Hematocrit; Plt: Platelet.
(DOCX)

S2 Table Summary of activated clotting times of heparinized whole blood samples. Blood was collected in sodium heparin vacutainer vacutainers (75 units/4mL; Becton Dickinson, Franklin Lakes) and tested immediately prior to perfusion experiments using using a Medtronic ACT-Plus system.
(DOCX)

S3 Table Comparison of cell line and primary cell thrombosis. Xenoperfusion on cell line and primary cell WT and GalTKO ± hCD46 endothelia was performed to validate thrombosis under different cell culture conditions. Primary cell endothelia displayed similar or more intense thrombosis than cell line endothelia. *All experiments were performed with heparinized blood. a.u., arbitrary units; TV, thrombus volume; Δ, relative change; SA, percent surface area coverage; FR, fluorescence ratio; T_50_, time to 50% maximal surface area coverage. All Δ & *P* values are versus respective cell line controls, and are expressed as mean ± SEM.
(DOCX)

S1 Video Xenothrombosis on wild type endothelium. A single WT endothelium perfused with heparinized human blood, demonstrating representative high-grade thrombosis. Images were taken at 30-second intervals for 50 minutes at 100x magnification using a 100-millisecond exposure. Source images were rendered at 11 frames per second using ImageJ.
(AVI)

S2 Video Four-dimensional representation of xenothrombosis on wild type endothelium. 3D surface renditions of the images from S1 Video were generated and combined using ImageJ to demonstrate xenothrombosis on WT endothelium in 4D (*x*, *y*, *z* and time). The video demonstrates high-grade thrombosis with extensive adhesion and aggregation.
(AVI)

S3 Video Xenothrombosis on GalTKO endothelium. A single, representative GalTKO endothelium perfused with heparinized human blood. Compared to thrombosis on WT endothelium, adhesion is reduced and delayed, resulting in less overall thrombosis. Images were taken at 30-second intervals for 50 minutes at 100x magnification using a 100-millisecond exposure. Source images were rendered at 11 frames per second using ImageJ.
(AVI)

S4 Video Four-dimensional representation of xenothrombosis on GalTKO endothelium. 3D surface renditions of the images from S3 Video were generated and combined using ImageJ to demonstrate xenothrombosis on GalTKO endothelium in 4D (*x*, *y*, *z* and time). The video demonstrates that although adhesion and total thrombus volume are decreased, aggregation remains relatively intact as demonstrated by the volumetric accumulation where thrombus is present.
(AVI)
