# Supplementary figures and images for: Four-Dimensional Characterization of Thrombosis in a Live-Cell, Shear-Flow Assay: Development and Application to Xenotransplantation
Source: PLoS One. 2015 Apr 1;10(4):e0123015. doi: 10.1371/journal.pone.0123015 (PMC4382176; doi:10.1371/journal.pone.0123015)

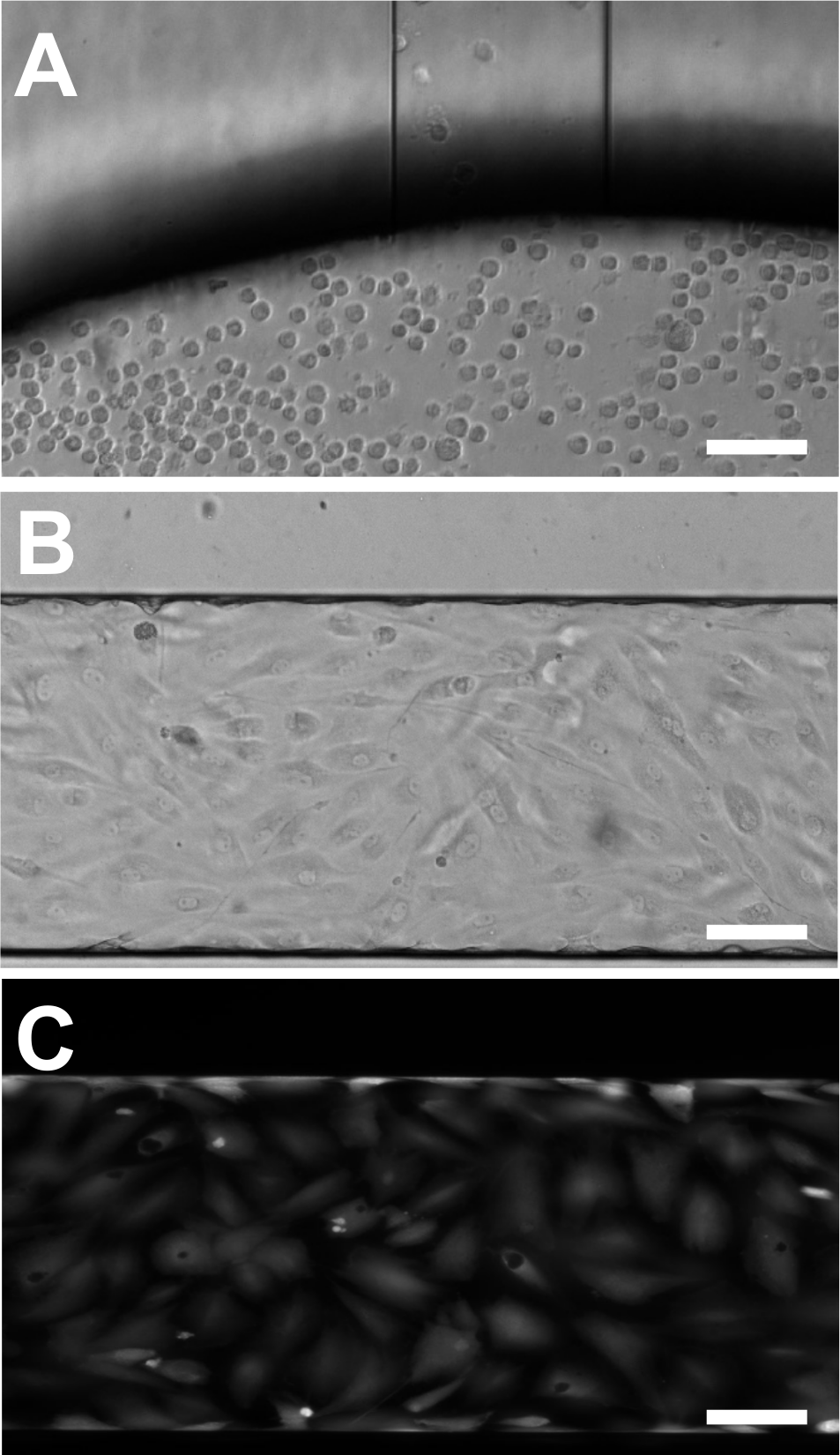

Supplement: S1 Fig — Endothelial cells are perfused from the inflow well (A) into the channel, where they are cultured to confluence over 48–72 hours (B & D). Scale bars: 100μm. (TIF) [file pone.0123015.s001.tif]

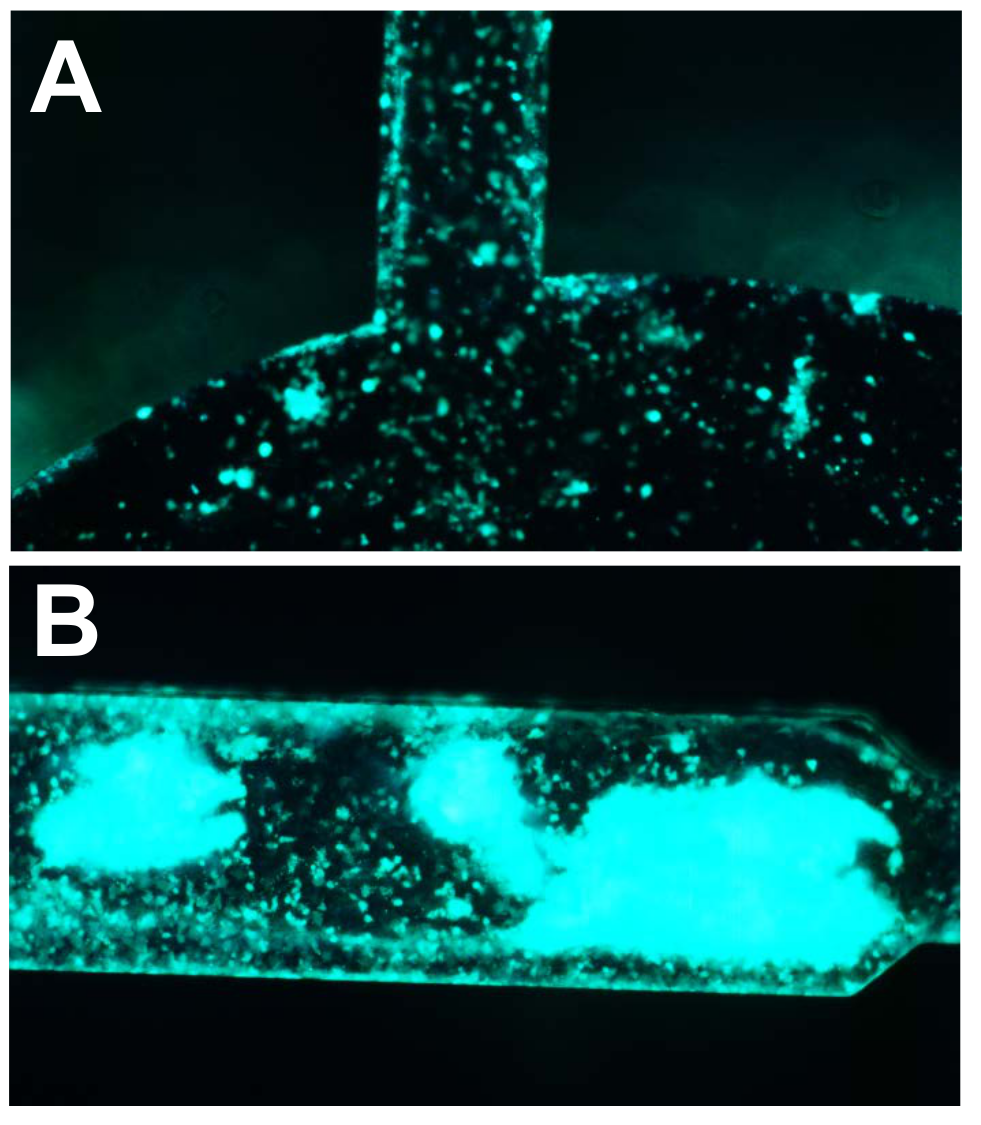

Supplement: S2 Fig — After 50 minutes of perfusion, blood remaining in the inflow well reservoir demonstrates no significant thrombosis (A), which is instead limited to the perfusion channel (B). (TIF) [file pone.0123015.s002.tif]
