# Supplementary material for: Four-Dimensional Characterization of Thrombosis in a Live-Cell, Shear-Flow Assay: Development and Application to Xenotransplantation
Source: PLoS One. 2015 Apr 1;10(4):e0123015. doi: 10.1371/journal.pone.0123015 (PMC4382176; doi:10.1371/journal.pone.0123015)
Supplement: S1 Table — Results were obtained using a Drew Scientific Hemavet automatic counter. WBC: white blood cells; Hgb: Hemoglobin; Hct: Hematocrit; Plt: Platelet. (DOCX) [file pone.0123015.s003.docx]

| **Sample** | **WBC, K/µL** | **Hgb, g/dL** | **Hct, %** | **Plt, K/µL** |
| --- | --- | --- | --- | --- |
| 130503 | 4.3 | 12.1 | 38.3 | 250.0 |
| 130505 | 4.6 | 12.2 | 38.2 | 238.0 |
| 130507 | 4.4 | 12.5 | 37.9 | 257.0 |
| 130508 | 4.0 | 12.2 | 39.9 | 256.0 |
| 130524 | 5.3 | 13.4 | 41.5 | 173.0 |
| 130610 | 3.7 | 12.2 | 38.2 | 224.0 |
| 130701 | 4.3 | 18.2 | 49.7 | 278.0 |
| 130731 | 4.1 | 12.3 | 36.8 | 180.0 |
| 130807 | 4.1 | 12.2 | 37.0 | 177.0 |
| 130912 | 4.9 | 14.2 | 44.6 | 254.0 |
| 131031 | 4.0 | 16.6 | 41.4 | 250.0 |
|  |  |  |  |  |
| Average ± SD | 4.3 ± 0.4 | 13.5 ± 2.0 | 40.3 ± 3.7 | 231 ± 35 |
|  |  |  |  |  |
| Reference | 4.0 – 11.0 | 12.0 – 16.0 | 35.0 – 45.0 | 150 - 450 |
