# Supplementary material for: Four-Dimensional Characterization of Thrombosis in a Live-Cell, Shear-Flow Assay: Development and Application to Xenotransplantation
Source: PLoS One. 2015 Apr 1;10(4):e0123015. doi: 10.1371/journal.pone.0123015 (PMC4382176; doi:10.1371/journal.pone.0123015)
Supplement: S2 Table — Blood was collected in sodium heparin vacutainer vacutainers (75 units/4mL; Becton Dickinson, Franklin Lakes) and tested immediately prior to perfusion experiments using using a Medtronic ACT-Plus system. (DOCX) [file pone.0123015.s004.docx]

| **Series** | **Activated Clotting Time, sec** |
| --- | --- |
| 130613 | >999 |
| 130701 | >999 |
| 130731 | >999 |
| 130809 | >999 |
| 131030 | >999 |
