# Supplementary material for: Four-Dimensional Characterization of Thrombosis in a Live-Cell, Shear-Flow Assay: Development and Application to Xenotransplantation
Source: PLoS One. 2015 Apr 1;10(4):e0123015. doi: 10.1371/journal.pone.0123015 (PMC4382176; doi:10.1371/journal.pone.0123015)
Supplement: S3 Table — Xenoperfusion on cell line and primary cell WT and GalTKO ± hCD46 endothelia was performed to validate thrombosis under different cell culture conditions. Primary cell endothelia displayed similar or more intense thrombosis than cell line endothelia. *All experiments were performed with heparinized blood. a.u., arbitrary units; TV, thrombus volume; Δ, relative change; SA, percent surface area coverage; FR, fluorescence ratio; T50, time to 50% maximal surface area coverage. All Δ & P values are versus respective cell line controls, and are expressed as mean ± SEM. (DOCX) [file pone.0123015.s005.docx]

| **Cells:** | **WT** | **WT** | **GalTKO** | **GalTKO.hCD46** |
| --- | --- | --- | --- | --- |
| **PAEC type:** | cell line | primary | cell line | primary |
| **Perfusate:** | human blood | human blood | human blood | human blood |
| **Treatment:*** | none | none | none | none |
|  |  |  |  |  |
| **N** | 48 | 10 | 11 | 10 |
|  |  |  |  |  |
| **Thrombus Volume (TV), a.u.** | 7831 ± 238 | 6073 ± 528 | 3098 ± 594 | 2019 ± 585 |
| Δ |  | -22% |  | -35% |
| *P* |  | 0.02 |  | 0.09 |
|  |  |  |  |  |
| **Adhesion (SA), %** | 65.0 ± 1.7 | 49.2 ± 4.1 | 27.5 ± 4.2 | 18.3 ± 4.2 |
| Δ |  | -24% |  | -33% |
| *P* |  | 0.008 |  | 0.14 |
|  |  |  |  |  |
| **Aggregation (FR), a.u.** | 120.0 ± 1.0 | 123.1 ± 1.4 | 107.0 ± 4.9 | 110.5 ± 7.9 |
| Δ |  | +3% |  | +3% |
| *P* |  | 0.02 |  | 0.75 |
|  |  |  |  |  |
| **Kinetics (T_50_), min.** | 21.9 ± 1.7 | 9.2 ± 2.7 | 31.0 ± 2.8 | 21.9 ± 2.4 |
| Δ |  | -58% |  | -28% |
| *P* |  | 0.002 |  | 0.07 |
